# Rapid disease progression during adjuvant chemotherapy following concurrent chemoradiotherapy and radical resection for rectal sarcomatoid carcinoma: a case report and literature review

**DOI:** 10.3389/fonc.2026.1946588

**Published:** 2026-10-28

**Authors:** Weiti Chen, Penghong Zeng, Huang Cao, Ziwei Zhang, Ting Jiang, Fei Ma, Meijing Chen, Di Li

**Affiliations:** 1 Department of General Surgery, Xiamen Susong Hospital, Xiamen, Fujian, China; 2 Puxi Elementary School, Shanghai American School, Shanghai, China; 3 Department of Radiology, Xiamen Susong Hospital, Xiamen, Fujian, China; 4 Department of Pathology, Xiamen Susong Hospital, Xiamen, Fujian, China

**Keywords:** chemoradiotherapy, colorectal, outcomes, sarcomatoid carcinoma, surgery

## Abstract

**Background:**

Rectal sarcomatoid carcinoma (RSC) is an exceedingly rare, aggressive malignancy characterized by a dismal prognosis, high recurrence rates, and truncated overall survival. Only 16 cases have been reported. Due to its rarity, optimal therapeutic strategies remain poorly understood. While current management typically mirrors rectal adenocarcinoma paradigms due to a lack of dedicated guidelines, clinical outcomes are frequently unsatisfactory, highlighting the challenges in managing this rare entity.

**Case description:**

A 66-year-old male presented with a 3-month history of hematochezia. Colonoscopy revealed a rectal mass, and subsequent biopsy confirmed a moderately-to-poorly differentiated adenocarcinoma(~95%), with only focal areas showing sarcomatoid differentiation(~5%). Immunohistochemistry (IHC) demonstrated proficient mismatch repair (pMMR) status and low HER-2 expression (1+). Pelvic MRI clinically staged the tumor as cT4aN2Mx (MRF-). Digital rectal examination palpated a hard, cauliflower-like mass 9 cm from the anal verge. On October 16, 2025, the patient initiated neoadjuvant chemoradiotherapy (CRT) (50 Gy/25 fractions with concurrent capecitabine), followed by two cycles of XELOX. On January 9, 2026, he underwent laparoscopic radical resection, right lateral pelvic lymph node dissection, and protective ileostomy. Postoperative pathology indicated downstaging to ypT3N0 with a tumor regression grade (TRG) of 2 (~70% overall regression). Notably, the residual tumor predominantly exhibited sarcomatoid differentiation(~70%) alongside intramucosal carcinoma components(~30%), confirmed as RSC via IHC. Postoperatively, the patient received four cycles of adjuvant XELOX(interrupted temporarily for stoma reversal following MDT evaluation). During adjuvant chemotherapy (May 10, 2026), he presented with significantly aggravated abdominal distension. Follow-up contrast-enhanced CT revealed right abdominal alterations highly suspicious of abscess or metastasis, with massive ascites and pelvic effusion. Cytological and IHC analyses of the ascites cell block confirmed peritoneal metastasis originating from the sarcomatoid component, showing focal positivity for CKpan, diffuse and strong positivity for Vimentin, and positivity for CDX2 while maintaining a pMMR/MSS status. Regrettably, the patient passed away on May 18, 2026.

**Conclusion:**

RSC is a rare, aggressive biphasic malignancy with a median overall survival typically under six months. It exhibits profound resistance to conventional colorectal CRT regimens, with a high propensity for recurrence and metastasis. Standard treatments are frequently inadequate, underscoring the urgent need for novel individualized therapeutic and targeted approaches.

## Introduction

Sarcomatoid carcinoma (SC) is a rare biphasic neoplasm characterized by the coexistence of epithelial and mesenchymal differentiation. According to the World Health Organization (WHO) classification, “colorectal carcinoma with sarcomatoid components” serves as the overarching diagnostic umbrella term for such entities, while “sarcomatoid carcinoma” and “carcinosarcoma” are often used to describe specific histological phenotypes or variants exhibiting biphasic features. First described by Virchow in 1864 (1), this malignancy predominantly arises in the female genital tract(41.10%) and respiratory tract (29.09%) (2), whereas rectal involvement is exceedingly rare. Clinically, SC is characterized by rapid tumor growth and early metastasis, with available literature suggesting an estimated median overall survival (OS) of less than six months for colorectal sarcomatoid carcinoma (CRSC)—an estimate largely derived from heterogeneous case reports and small series rather than a uniform cohort (3, 4). In recent years, its incidence and prevalence have continued to rise (2). Based on our literature review, primary rectal sarcomatoid carcinoma (RSC) is exceptionally rare, with only 16 cases reported to date. Crucially, there has been no published case report of a patient who underwent the structured sequence of a comprehensive regimen comprising neoadjuvant concurrent chemoradiotherapy (CRT), radical surgery, and adjuvant chemotherapy. Herein, we report a rare case of RSC in which the patient developed peritoneal carcinomatosis and rapidly succumbed to the disease despite receiving this systematic multimodal treatment.

## Case report

A 66-year-old male presented with a three-month history of hematochezia. Colonoscopy revealed a rectal mass, and subsequent biopsy confirmed a predominantly moderately-to-poorly differentiated adenocarcinoma(95%), with only focal areas showing sarcomatoid differentiated (5%)(**Figures 1A, B**). Immunohistochemical (IHC) staining demonstrated intact expression of mismatch repair proteins (MSH2+, MSH6+, MLH1+, PMS2+), indicating a proficient mismatch repair/microsatellite stable (pMMR/MSS) status. HER-2 expression was low (1+). Pelvic magnetic resonance imaging (MRI) performed on October 13, 2025 (**Figure 2A**), indicated a locally advanced tumor in the middle-to-upper rectum (cT4aN2Mx) with no involvement of the mesorectal fascia (MRF-). Digital rectal examination (DRE) in the left lateral decubitus position revealed a hard, cauliflower-like mass at the 3 o’clock position, located approximately 9 cm from the anal verge, with normal anal sphincter tone and no gross blood on the examining glove upon withdrawal.

**Figure 1:**
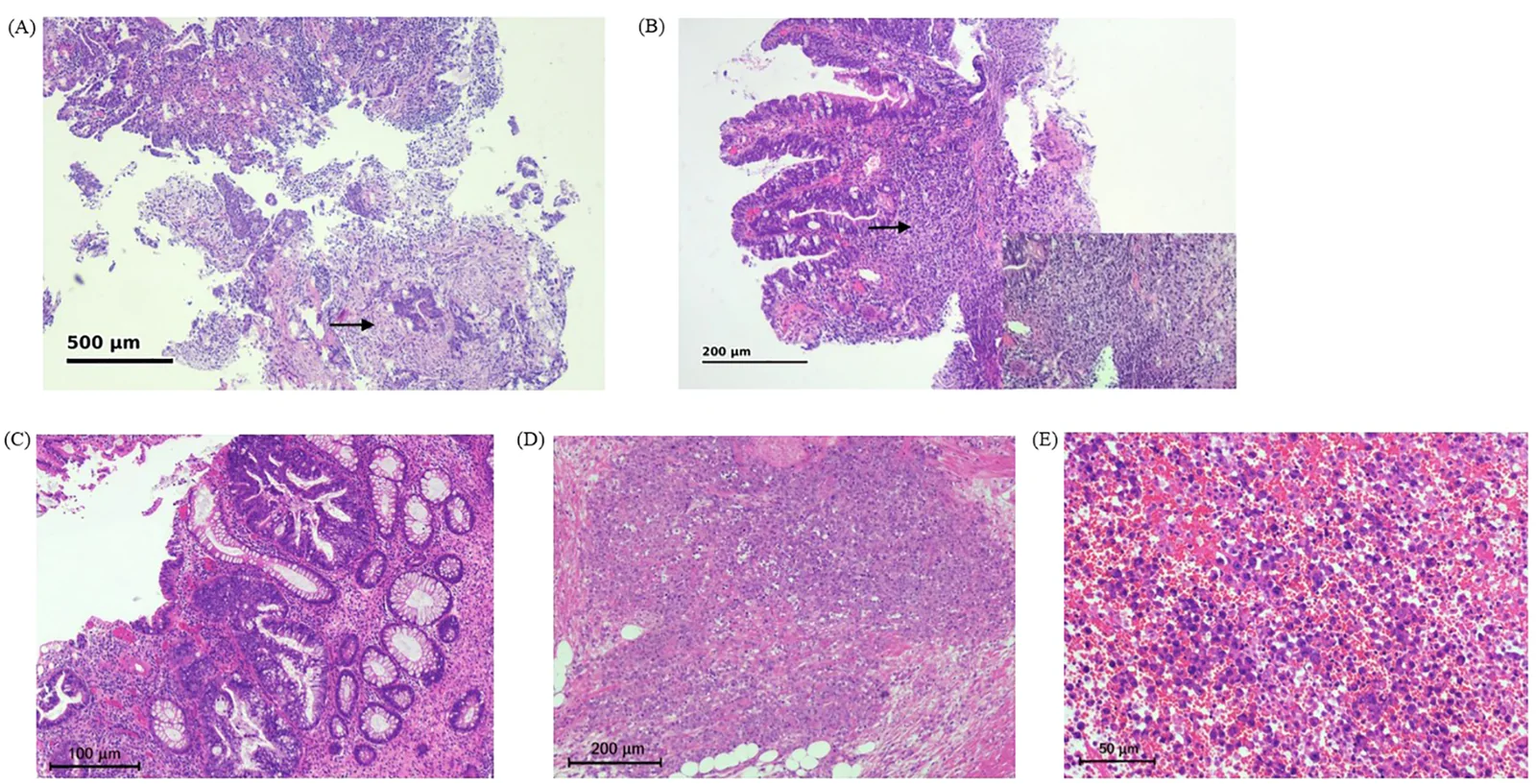
Pathological and cytological findings of the patient’s biopsies, surgical resection specimen, and ascitic fluid cell block (H&E staining). **(A)** Biopsy specimen showing the moderately differentiated adenocarcinoma component, characterized by dysplastic glands infiltrating the stroma with a desmoplastic reaction (HE, ×40). **(B)** Biopsy specimen showing the sarcomatoid component with a clear demarcation from the adenocarcinoma area, featuring sheet-like arrangements of spindle/epithelioid cells with marked atypia infiltrating the stroma (HE, ×100; inset shows higher magnification at ×200). **(C)** Resected specimen showing the intramucosal carcinoma component in the residual tumor bed, featuring papillary/cribriform proliferation of atypical epithelial glands without basement membrane destruction or stromal invasion, demonstrating marked regression compared to the pre-treatment invasive adenocarcinoma (HE, ×200). **(D)** Resected specimen showing the sarcomatoid component in the residual tumor bed, featuring sheet-like distribution of epithelioid/spindle cells invading the bowel wall with severe nuclear atypia, vesicular nuclei, prominent nucleoli, and scattered apoptotic/necrotic foci amid stromal edema and fibroplasia, showing persistent components with limited regression compared to the initial biopsy (HE, ×100). **(E)** Cell block section of the ascitic fluid showing cohesive sheets of malignant tumor cells with severe atypia, vesicular nuclei, prominent nucleoli, and pathological mitotic figures, closely resembling the morphology of the sarcomatoid components observed in the biopsy and resection specimens (HE, ×200).

**Figure 2:**
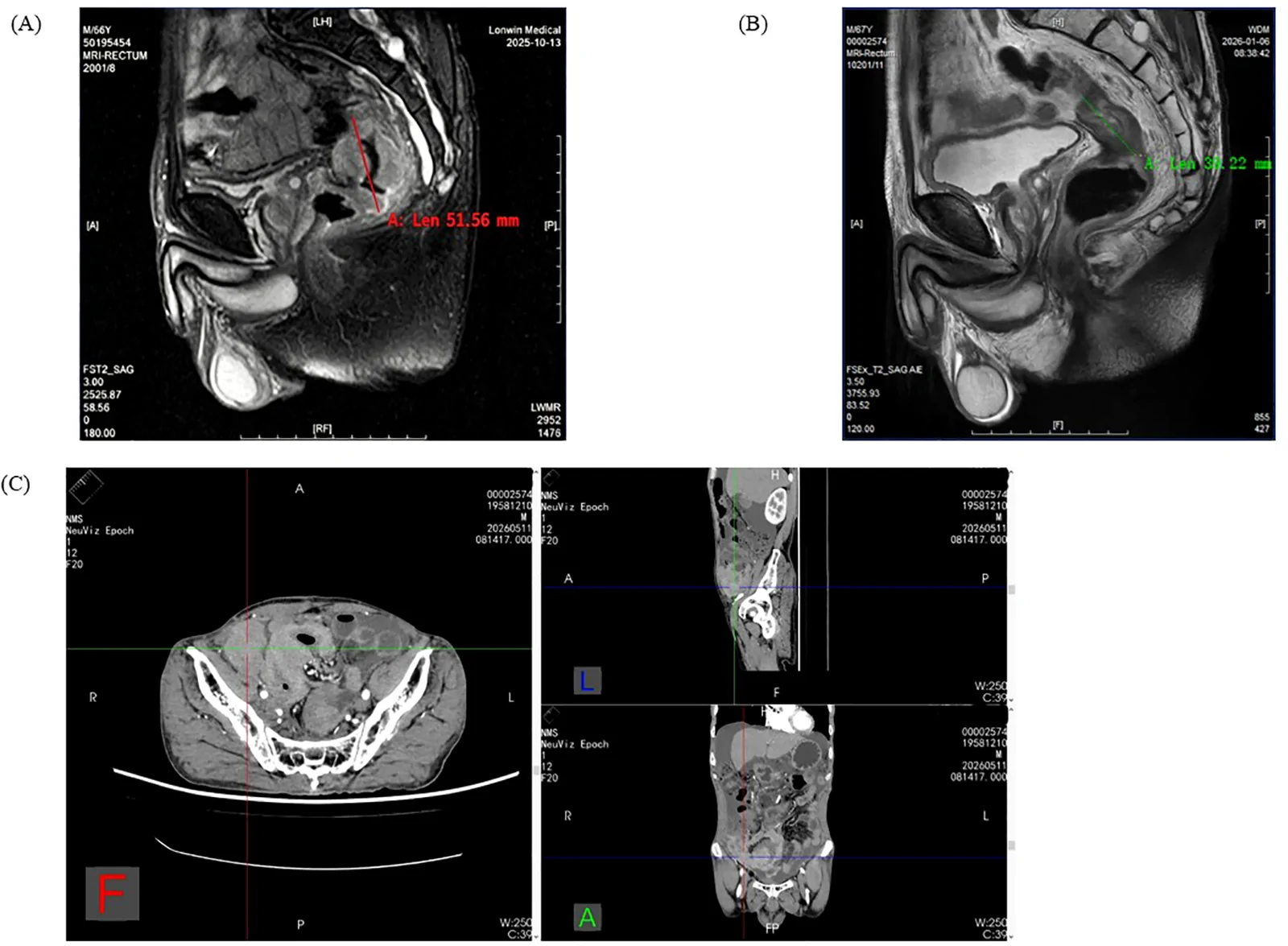
Results of pelvic Magnetic Resonance Imaging(MRI) before and after radiotherapy as well as follow-up contrast-enhanced computed tomography (CT). **(A)** Sagittal view of pre-radiotherapy pelvic MRI scan. **(B)** Sagittal view of MRI scan of the pelvis after radiotherapy, revealing significant tumor regression compared to the baseline scan. **(C)** CT scan demonstrating abdominal metastases and massive ascites.

On October 16, 2025, the patient initiated CRT, receiving a total radiation dose of 50 Gy in 25 fractions, alongside oral capecitabine (1.5 g twice daily). Following CRT, during the standard interval for radiation recovery and tumor downstaging evaluation, he received two cycles of consolidation XELOX chemotherapy on November 26 and December 17, 2025, acting as an MDT-guided bridging strategy prior to surgical re-evaluation (**Figure 2B**). On January 9, 2026, the patient underwent laparoscopic radical proctectomy, right lateral pelvic lymph node dissection (LPLND), and a protective ileostomy under general anesthesia.

Pathological examination of the resected specimen revealed an ulcerated tumor measuring 2.0 × 1.8 × 0.8 cm in the lower rectum. The tumor exhibited substantial regression following neoadjuvant therapy, with the tumor bed showing interstitial edema, a desmoplastic reaction, and infiltration of lymphocytes and histiocytes, corresponding to a Tumor Regression Grade(TRG) of 2(defined as residual cancer cells with partial tumor regression) Notably, the residual tumor predominantly displayed sarcomatoid differentiation (accounting for ~70%), while the overlying mucosa contained intramucosal carcinoma components(accounting for ~30%) (**Figures 1C, D**). The tumor invaded the sub serosal layer (ypT3) with high-grade tumor budding (>10 buds/0.785 mm^2^, Bd3). No lymph vascular or perineural invasion was identified (vascular invasion pending elastic fiber staining). All resection margins (including the circumferential, proximal, and distal margins) were clear, and no lymph node metastasis was found in the regional pericolic lymph nodes (0/18, ypN0). The right lateral pelvic lymph nodes were also negative (0/2). The final pathological staging was ypT3N0Mx. (Note: The TRG system follows the AJCC criteria: TRG0, complete response; TRG1, near complete response; TRG2, partial response with residual cancer cells; TRG3, poor or no response).

IHC analysis of the resected specimen demonstrated that both the intramucosal carcinoma and sarcomatoid components maintained proficient mismatch repair (pMMR) status. Specifically, the intramucosal carcinoma component showed diffuse and strong positivity for CK20, CDX-2, CKpan, SATB2, CK8/18, and EMA, whereas the sarcomatoid component showed diffuse and strong positivity for Vimentin, alongside weak positivity for CK20, CDX-2, CKpan, SATB2, CK8/18, and EMA. Both components exhibited complete loss of p53 expression (negative), while CEA, S100, CD34, SMA, Syn, and CgA were all negative. These comprehensive IHC findings, combined with the clinical history, definitively confirmed the diagnosis of residual SC with treatment response.

Postoperatively, the patient initiated adjuvant XELOX chemotherapy on February 3, 2026, receiving intravenous oxaliplatin (160 mg) combined with split-dose capecitabine (1.0 g in the morning and 1.5 g in the evening). On March 2, 2026, he underwent laparoscopic exploration and ileostomy reversal under general anesthesia, which temporarily interrupted the continuity of systemic chemotherapy. Although our institutional protocol prioritizes completing the full course of adjuvant chemotherapy prior to stoma reversal, this pragmatic deviation was necessitated by the patient’s severe psychological distress regarding the stoma and his firm refusal of further treatment without prior reveral, as agreed upon following a multidisciplinary evaluation to ensure overall clinical compliance. This was followed by three additional cycles of the identical XELOX regimen on March 19, April 9, and April 30, 2026.

During the subsequent clinical follow-up, on May 6, 2026, the patient was readmitted with localized right lower quadrant abdominal pain and discomfort. Contrast-enhanced computed tomography (CT) revealed thickening of the right lower abdominal bowel wall and a trace amount of peritoneal effusion. Despite supportive care—including anti-infective therapy, fluid resuscitation, and nutritional support—which initially provided transient symptomatic relief, his abdominal distension worsened significantly by May 10. A repeat CT scan on May 11 showed right-sided abdominal changes (highly suspicious for either an abscess or metastasis/tumor recurrence) and massive abdomino pelvic accumulation (**Figure 2C**). Consequently, a percutaneous peritoneal catheter drainage yielded hemorrhagic ascites. Cytological and IHC analysis of the cell block derived from the ascitic fluid revealed numerous malignant tumor cells (**Figure 1E**), with IHC showing focal positivity for CKpan, diffuse and strong positivity for Vimentin, and positivity for CDX2,while CK7, villin, CD45, CD56, Syn, CgA, and S100 were all negative. This profile confirmed peritoneal metastasis originating from the sarcomatoid component of the RSC. Due to this rapid and fulminant disease progression, the patient’s performance status deteriorated significantly(ECOG score of 4), rendering him completely intolerant to any further second-line chemotherapy or targeted therapy. Reflecting the patient’s and family’s shared perspective under terminal circumstances, they declined further aggressive interventions and requested voluntary discharge for palliative care, with the final follow-up concluding as the patient ultimately succumbed to the disease on May 18, 2026 (**Figure 3**).

**Figure 3:**
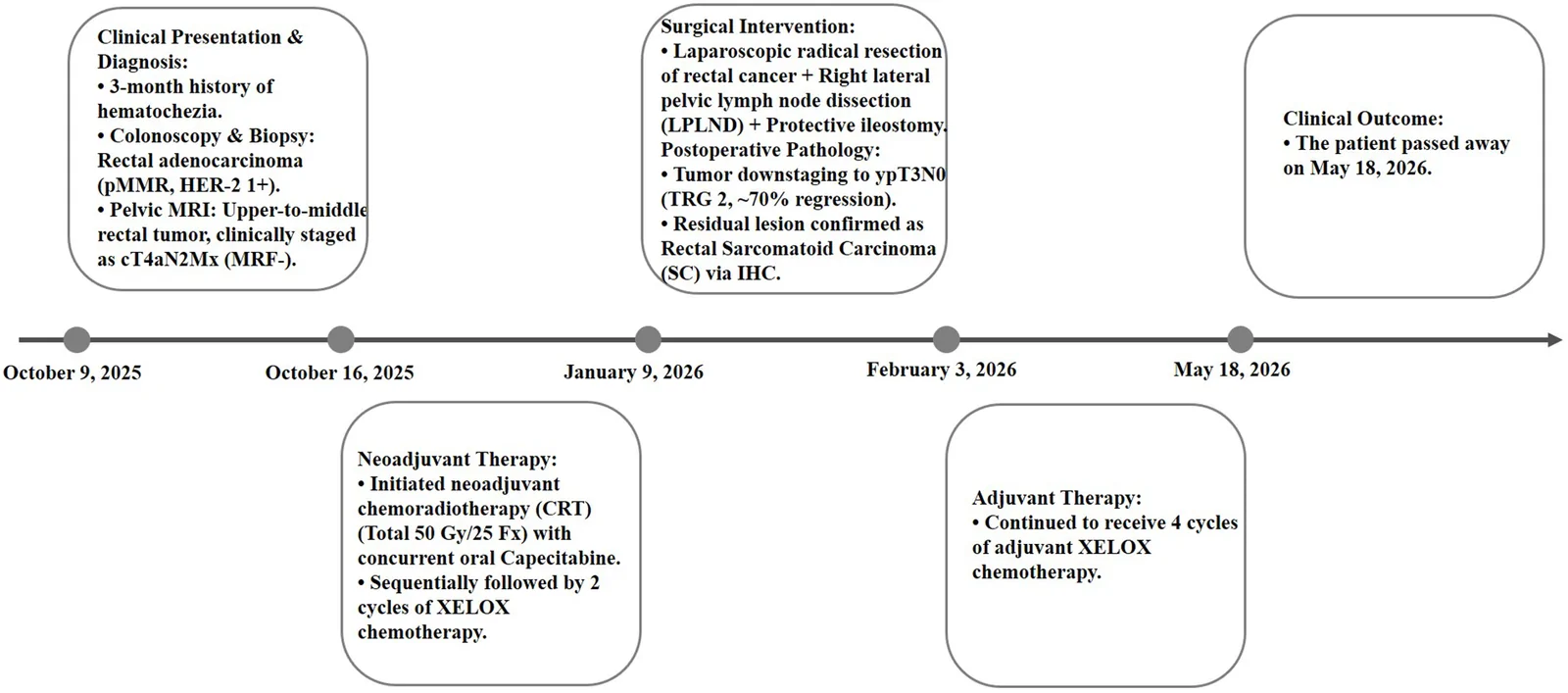
Timeline of clinical events.

## Discussion

RSC is a rare, highly aggressive malignancy characterized by a biphasic differentiation profile exhibiting both malignant epithelial and mesenchymal components. It falls under the category of colorectal carcinoma with sarcomatoid components, a tumor entity also referred to as carcinosarcoma (19). Despite its morphological appearance of sarcomatoid features (e.g., spindle cells and giant cells), its biological nature is that of an epithelium-derived carcinoma that acquires a mesenchymal-like morphology through epithelial–mesenchymal transition (EMT). This mechanism explains the biphasic morphology encompassing both carcinomatous and sarcomatoid components observed in this tumor.

Regarding the molecular evidence of its origin, TP53 mutations are closely associated with this phenotypic transition from carcinoma to sarcoma (16). Genomic studies on pulmonary sarcomatoid carcinoma (PSC) have demonstrated that the vast majority of cases (97%) share core genomic alterations between the carcinomatous and sarcomatoid components, indicating a common origin (5). Although no paired molecular sequencing was performed in the present patient to directly confirm clonal evolution, literature suggests that the sarcomatoid component typically rather arises from the primordial carcinomatous component via EMT rather than originating independently (20).

RSC is an exceedingly rare malignancy in which reported individual cases indicate an estimated median overall survival of less than six months, though this is drawn from heterogeneous colorectal sarcomatoid carcinoma literature. In our case, the patient’s survival from initial diagnosis to death was approximately 4 months, closely aligning with these previously documented aggressive clinical courses. Due to the scarcity of published literature and the total absence of evidence-based treatment guidelines, current management strategies predominantly rely on empirical regimens established for colorectal adenocarcinoma. Furthermore, existing literature primarily focuses on CRSC as a broad entity, lacking studies dedicated exclusively to the rectal subtype. To address this gap, this study compiled 16 previously reported case reports of RSC. A comprehensive review of these characteristics(totaling 17 patients when including the present case, as summarized in **Table 1**) revealed a mean age at presentation of 65 years, with a female predominance of 52.9%(9/17). The most common presenting symptom was fresh hematochezia, followed by abdominal pain. Although the clinical manifestations of RSC mirror those of standard rectal adenocarcinoma, its prognosis is distinctly dismal; most patients ultimately develop distant metastases and succumb to the disease rapidly.

**Table 1:** Summary of rectal sarcomatoid carcinoma cases from the literature and the present case.

Case	Author,yr	Age(yr)	Sex	Surgery	Radiotherapy	Chemotherapy	Distant metastasis(with/without)	Outcome
1	Roncaroli, 1995 (5)	71	F	Yes	Yes	On	With	6mo DOD
2	Takeyoshi I,2000 (6)	82	M	Yes	No	No	With	6 mo, DOD
3	Lee JK,2008 (7)	52	F	Yes	No	No	Without	8mo,disease-free
4	Macaigne,2004 (8)	67	F	Yes	No	Yes	With	2 mo, DOD
5	Öztürk,2006 (9)	65	F	Yes	No	No	Without	Alive at 60 month follow up
6	Kolodziejzak, 2013 (10)	83	M	Unresectable	No	No	Without	5 weeks,DOD
7	Osholowu, 2017	68	M	–	–	Yes	With	–
8	Keramidaris,2012 (11)	78	M	Yes	–	–	With	–
9	Shah S,2001 (12)	57	M	Yes	–	–	With	5 mo, DOD
10	Ishida H,2003 (13)	80	F	Yes	–	–	–	6 months (DOD)
11	Tsekouras DK,2006	60	M	Yes	No	Yes	With	6 months (DOD)
12	Jeong YJ,2008 (14)	13	F	Yes	No	Yes	Without	Alive at 2-month follow-up
13	A Sudlow,2015 (15)	80	F	Yes	Yes	NO	With	25 months (DOD)
14	M Todori,2023 (16)	73	F	Yes	No	No	Without	Alive at 4-month follow-up
15	El Rhaoussi FZ,2026 (17)	64	F	NO	Yes	YES	With	lost to follow-up
16	Yuki Meda,2022 (18)	46	M	Yes	Yes	Yes	With	alive 12 months after the surgery
17	Present case	66	M	Yes	Yes	Yes	With	4 months (DOD)

DOD, died of disease.

(Note: Case 1 to 16 are derived from previous literature, and Case 17 is the present case.).

From diagnostic reasoning to definitive pathology, histologically, RSC typically comprises two distinct pathological components: adenocarcinoma and SC. The epithelial component usually presents as high-grade adenocarcinoma, while the sarcomatoid component typically consists of spindle, pleomorphic, or anaplastic cells located deep within the tumor (15, 21). Clinically, diagnostic reasoning often begins with standard adenocarcinoma on superficial biopsy, which can mask the underlying sarcomatoid elements; consequently, inadequate biopsy depth or insufficient tissue sampling can easily lead to a missed diagnosis (7).

Given its composition of epithelial and mesenchymal cells, this tumor theoretically disseminates via hematogenous, lymphatic, peritoneal and serosal routes, explaining the patient’s peritoneal seeding. The biphasic nature of this tumor is a critical clinical feature, as superficial or inadequate biopsies may miss vital tumor components, thereby impeding accurate diagnosis and subsequent therapeutic management (22).

In the present case, the patient initially presented to our hospital with hematochezia. Colonoscopy revealed a rectal mass, and the endoscopic biopsy indicated a moderately-to-poorly differentiated adenocarcinoma; consequently, a treatment regimen for rectal adenocarcinoma was initially initiated. Preoperative clinical staging revealed a cT4aN2Mx rectal tumor with a negative mesorectal fascia (MRF). The patient received standard neoadjuvant chemoradiotherapy followed by two cycles of XELOX chemotherapy, and subsequently underwent laparoscopic radical resection of the rectum, right-sided LPLND, and temporary ileostomy. Postoperative pathology demonstrated substantial tumor regression (approximately 70%), with the residual lesions predominantly consisting of SC (ypN0). The final pathological staging was confirmed as ypT3N0Mx.

The definitive diagnosis of RSC was established only after the postoperative pathological evaluation. Although the patient achieved substantial overall tumor regression following neoadjuvant chemoradiotherapy, the residual lesion was predominantly composed of the sarcomatoid component (~70%). While previous isolated reports have noted tumor progression during radiation (5, 17), attributing this solely to generalized radio-resistance requires caution. Rather than universal radio-resistance, the persistence of the sarcomatoid component may reflect intra tumoral heterogeneity and the selective survival of pre-existing treatment-resistant sub-clones, compounded by the intrinsically aggressive biology of the tumor.

Postoperatively, the patient received four cycles of the XELOX regimen. However, malignant ascites developed during the final cycle of chemotherapy, accompanied by rapid disease progression and distant metastases, which ultimately led to the patient’s swift demise. Yuki Med et al. (18) also reported a male patient who underwent abdominoperineal resection for stage IIIb rectal cancer (cT3N2M0). Despite receiving seven cycles of XELOX (capecitabine, oxaliplatin, and leucovorin), the patient experienced local recurrence. While these observations in individual cases highlight the clinical challenge, declaring a universal insensitivity of RSC to oxaliplatin and capecitabine based solely on single-patient outcomes is premature. Instead, disease progression under treatment in this case is more likely attributable to intra tumoral heterogeneity, the selective proliferation of resistant sub-clones, and the intrinsically aggressive nature of the disease, warranting broader validation through *in vitro* cellular assays, animal models, and multicenter studies with large sample sizes.

Currently, proposed treatment regimens include 5-fluorouracil, leucovorin, doxorubicin, and cisplatin, with radiotherapy utilized as an adjunctive modality. Nevertheless, there is still no definite evidence demonstrating that these approaches can tangibly improve overall survival in such patients (9, 21, 23). In our case, the patient succumbed to the disease four months postoperatively, a clinical course that aligns closely with the median survival time reported in the existing literature (24).

Traditionally, surgical resection has been considered the mainstay of treatment for RSC. However, our literature review indicates that despite undergoing surgery, many patients experience a short survival time. A retrospective study by Dante L. S. Souza et al. (24) demonstrated that median OS for CRSC was merely 3.5 months, with no statistically significant difference in survival rates between patients who underwent surgery and those who did not. Notably, patients with stage I disease achieved a survival time exceeding 48 months, whereas those with stage II and III disease survived for only 3 to 13 months. For stage I RSC, radical surgery yields a favorable prognosis without the necessity for adjuvant chemoradiotherapy, which is consistent with findings in other studies (7, 9). However, once the tumor invades the muscularis propria or develops lymph node metastasis, surgical resection alone is rarely curative (3, 24). Given the limited efficacy of conventional radiotherapy and chemotherapy, there is an urgent need to explore targeted therapies and immunotherapies. Currently, both carcinomas with sarcomatoid components and undifferentiated carcinomas are recognized as highly aggressive malignancies lacking targeted therapeutic options (25).

In our case, IHC analysis revealed proficient mismatch repair (pMMR/MSS) status and ultra-low HER2 expression, findings that align with previous literature. Yuki Med et al. (18) reported a case of RSC with local postoperative recurrence where the patient survived 12 months following the administration of bevacizumab. Similarly, PSC is also known to be insensitive to conventional chemotherapy and radiotherapy. Due to its high mutation burden and significant immune infiltration, targeted and immunotherapies are considered highly promising for PSC. Clinical studies in PSC have demonstrated that anti-angiogenic agents combined with chemotherapy yield significantly superior efficacy compared to chemotherapy alone. Furthermore, the application of various bispecific antibodies (e.g., PD-1/VEGF or PD-L1/VEGF dual-targeting agents) also warrants clinical investigation in PSC.

Epithelial-derived carcinomas can acquire a mesenchymal-like morphology through EMT, thereby transforming into sarcomatoid carcinomas. Savolitinib is currently the only MET inhibitor that has been prospectively investigated in patients harboring MET exon 14 skipping mutations, and it has been shown to improve the therapeutic outcomes in PSC (26). Whether this agent can be similarly applied to RSC remains to be determined, as there is currently a lack of literature on this topic.

According to previously reported literature, the clinical severity of this disease is often underestimated; distant metastases are frequently discovered intraoperatively or during short-term postoperative follow-up. Current management strategies typically follow the clinical pathways established for rectal adenocarcinoma. However, while radical surgery yields favorable outcomes for stage I patients, traditional surgery, radiotherapy, and chemotherapy demonstrate limited efficacy for patients at stage II and beyond. Given that current clinical evidence is predominantly based on sporadic case reports and small retrospective series, our case demonstrates the sobering possibility of rapid progression and resistance despite comprehensive multimodal treatment, rather than proving universal treatment failure in all RSC cases. These observations suggest that relying solely on conventional adenocarcinoma paradigms may be insufficient for advanced RSC, thereby emphasizing the urgent need for individualized therapeutic strategies based on a deeper understanding of its biology.

## Conclusions

RSC is an exceedingly rare biphasic malignancy characterized by a dismal prognosis. Reflecting historical data from heterogeneous colorectal sarcomatoid carcinoma literature and our present case, the median overall survival for RSC is generally estimated at under six months. Accumulating evidence suggests that advanced RSC is often associated with limited responsiveness to conventional chemoradiotherapy regimens used for colorectal cancer, alongside a high propensity for rapid recurrence and metastasis. Given the limitations observed in current literature and individual cases like ours, future therapeutic strategies should look beyond traditional paradigms, incorporating molecular profiling to explore novel targeted and immunotherapeutic avenues.

## Data Availability

The original contributions presented in the study are included in the article/**Supplementary Material**. Further inquiries can be directed to the corresponding author.
